# Mechanisms of Actions Involved in The Antinociceptive Effect of Estragole and its *β*-Cyclodextrin Inclusion Complex in Animal Models

**DOI:** 10.3390/plants11212854

**Published:** 2022-10-26

**Authors:** Roger Henrique Sousa da Costa, Anita Oliveira Brito Pereira Bezerra Martins, Renata Torres Pessoa, Saad Ali Alshehri, Shadma Wahab, Md Faruque Ahmad, Muath Suliman, Lucas Yure Santos da Silva, Isabel Sousa Alcântara, Andreza Guedes Barbosa Ramos, Maria Rayane Correia de Oliveira, Francisco Lucas Alves Batista, Gyllyandeson de Araújo Delmondes, Pablo Antonio Maia de Farias, Janaína Esmeraldo Rocha, Henrique Douglas Melo Coutinho, António Raposo, Conrado Carrascosa, José Raduan Jaber, Irwin Rose Alencar de Menezes

**Affiliations:** 1 Laboratory of Pharmacology and Molecular Chemistry (LFQM), Department of Biological Chemistry, Regional University of Cariri-URCA, Pimenta 63.100-000, Ceará, Brazil; 2 Department of Pharmacognosy, College of Pharmacy, King Khalid University, Abha 61421, Saudi Arabia,; 3 Department of Clinical Nutrition, College of Applied Medical Sciences, Jazan University, Jazan 45142, Saudi Arabia; 4 Department of Clinical Laboratory Sciences, College of Applied Medical Sciences, King Khalid University, Abha 61421, Saudi Arabia; 5 Graduate Program in Biotechnology-Northeast Biotechnology Network (RENORBIO), State University of Ceará (UECE), Fortaleza 60741-000, Ceará, Brazil; 6 Nursing Academic Collegiate, Federal University of Vale do São Francisco (UNIVASF), Petrolina 56330-400, Pernambuco, Brazil; 7 CECAPE College, Juazeiro do Norte-CE 63.024-015, Ceará, Brazil; 8 Laboratory of Microbiology and Molecular Biology, Department of Biological Chemistry, Regional University of Cariri-URCA, Pimenta 63.100-000, Ceará, Brazil; 9 CBIOS (Research Center for Biosciences and Health Technologies), Universidade Lusófona de Humanidades e Tecnologias, Campo Grande 376, 1749-024 Lisboa, Portugal; 10 Department of Animal Pathology and Production, Bromatology and Food Technology, Faculty of Veterinary, Universidad de Las Palmas de Gran Canaria, Trasmontaña s/n, 35413 Arucas, Spain; 11 Departamento de Morfologia, Facultad de Veterinaria, Universidad de Las Palmas de Gran Canaria, 35413 Las Palmas de Gran Canaria, Spain

**Keywords:** estragole, monoterpene, nociception, cyclodextrins

## Abstract

(1) Background: estragole is a monoterpene found in the essential oils of several aromatic plants, which can be used for several pharmacological activities. The aim of this study was to evaluate the antinociceptive effect of estragole (Es) and its *β*-cyclodextrins inclusion complex (Es/β-CD). (2) Methods: the effects of Es and Es/β-CD on the central nervous system (CNS) were evaluated through open field and rota-rod assays, and the antinociceptive effect in formalin models, abdominal writhing induced by acetic acid, hot plate, tail flick test and plantar mechanical hyperalgesia. (3) Results: Es and Es/β-CD showed no alterations on the CNS evaluated parameters and the results suggested there was an antinociceptive action in the formalin, abdominal writhing, hot plate, tail flick tests and plantar mechanical hyperalgesia, proposing the involvement of the nitric oxide, glutamatergic signaling pathways, cyclic guanosine monophosphate and vanilloid pathways. (4) Conclusion: the results suggest that Es and Es/β-CD have a promising antinociceptive potential as a possible alternative for the pharmacological treatment of pain, also showing that the encapsulation of Es in β-cyclodextrins probably improves its pharmacological properties, since the complexation process involves much lower amounts of the compound, contributing to better bioavailability and a lower probability of adverse effect development.

## 1. Introduction

Pain is a signal resulting from noxious stimuli, which tries to restore homeostasis in the affected tissues, activating reactions with the purpose of suppressing the etiology and, ultimately, limiting or recovering the lesions and disorders [1]. It is also considered an adaptive response aimed to maintain the integrity of the organism in the presence of stimuli that can generate tissue damage [2].

However, nociception is a sensory process stimulated through sensitization and activation of receptors, called nociceptors [3,4]. Pain and nociception are considered different terms; however, pain cannot occur without nociception, a process that involves the detection of stimuli that can be harmful, with consequent defensive and immediate reflex behavior [5]. The search for new drugs as therapeutic alternatives for pain treatment is a continuous one [6]. Most new drugs available on the market originate from natural products or compounds derived from natural products, especially those extracted from plants [7].

Terpenes are considered as one of the largest families of natural products and important precursors for biotechnological production [8,9] and are regarded as one of the largest classes of secondary metabolites [10]. The monoterpenes that are part of the terpene group are widespread in nature, especially in plants rich in essential oils, where they represent the main class regarding the composition of these oils, constituting 90% of them and showing several pharmacological activities [11], such as anti-inflammatory, antioxidant, antitumor, cardioprotective, and neuroprotective effects, among others [12].

Estragole (Es) is one of the most representative monoterpenes of the essential oils of aromatic plants of the genera *Croton, Artemisia, Ocimum, Illicium* and *Foeniculum*, showing antioxidant and antimicrobial pharmacological properties [13], anti-inflammatory activity, being pharmacologically potent and effective in oral doses lower than those considered to be toxic [14,15].

However, estragole, like most terpenoids, has limitations for its technological application due to its high volatility, instability to light, heat, as well as instability in the presence of oxygen, in addition to low aqueous solubility [16]. Therefore, to minimize these limitations, techniques of monoterpene inclusion in cyclodextrin molecules have been used, which is already common in the pharmaceutical industry [17], seeking to improve the bioavailability of these substances through encapsulation [18,19].

The aim of the present study was to assess the antinociceptive activity of estragole and its β-CD complex, as well as to investigate the possible signaling pathways involved in the antinociceptive response in animal models.

## 2. Results

### 2.1. Characterization of Inclusion Complexes

#### Es/β-CD Infrared Spectroscopy Measurement

**Figure 1:**
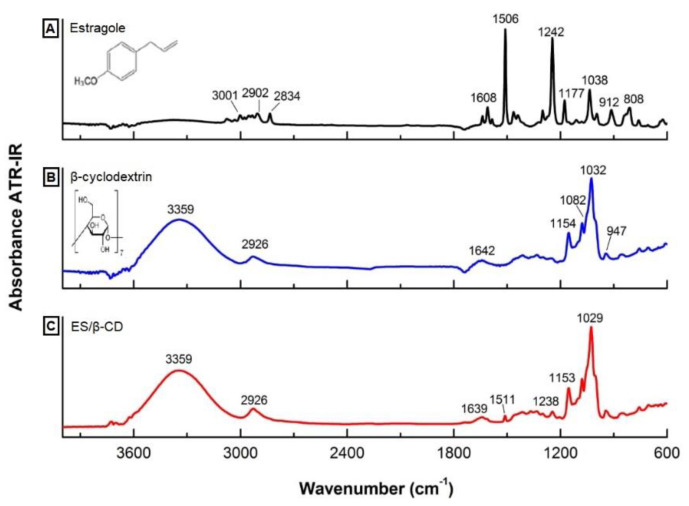
Spectrum in the infrared region: (**A**) estragole; (**B**) β-cyclodextrin; (**C**) Es/β-CD.

Attenuated total reflection infrared spectra (ATR-FTIR) showed common and specific bands of Es/β-CD, β-cyclodextrin and estragole, shown in Figure 1A–C. This characterization allowed us to obtain important information to confirm the identity of the analyzed substances, as in the identification of functional groups. Based on the analysis of the absorbance spectrum, characteristic bands of the estragole were identified; the mode evidenced at 3001 cm^−1^ correspond to the stretching of single bonds between carbon and hydrogen (C-H) that belong to the aromatic ring and weak bands in the region of 2800–2900 cm^−1^ may indicate axial stretching of the CH group bond. Located in the range of 1600–1400 cm^−1^ are the vibrational modes of double bond (C=C) of the ring; in the infrared spectrum of the estragole, these vibrations were identified at 1608 cm^−1^ and 1506 cm^−1^. The high intensity band observed at 1242 cm^−1^ is a single bond vibration between C-O-C and the wavenumber of 808 cm^−1^ is attributed to an angular deformation of the aromatic ring.

The *β*-cyclodextrin absorption spectrum showed characteristic bands of this substance, and it is possible to observe a typical stretching broad band of the hydroxyl group (O-H) in the wavenumber 3359 cm^−1^; the band evidenced at 2926 cm^−1^ is a stretching of the (C-H). The wavenumber 1642 cm^−1^ is an angular deformation between H-O-H, and in the region of 1260–1000 cm^−1^ are the stretching vibrations of the carbon and hydrogen bond groups; in the spectrum, this vibrational mode was observed in the bands at 1154 cm^−1^ (C-O stretching) and at 1032 cm^−1^ (C-O-C stretching).

In the Es/β-CD complex spectrum, characteristic peaks and a strong similarity with the β-CD spectrum were observed. In particular, the bands 3359, 2926, 1153 and 1029 cm^−1^ were very evident in the formed complex, with emphasis also on the bands 1511 cm^−1^ and 1238 cm^−1^, characteristic of the estragole, which showed a reduction in intensity when compared to the isolated spectrum (1505 cm^−1^ and 1242 cm^−1^, respectively), which had a small wavenumber shift, suggesting that these bonds are possibly involved in the formation of these complexes (Figure 1).

### 2.2. Evaluation of Estragole and Es/β-CD on the CNS

#### 2.2.1. Rota-Rod Test

In the rota-rod assay, after the treatment with Es and Es/β-CD at a dose of 60 mg/kg, the animals did not show characteristics of motor discoordination when compared to the control group, as the number of falls and permanence on the bar were similar.

#### 2.2.2. Open Field Test

Treatment with Es and Es/β-CD at a dose of 60 mg/kg did not reduce the number of behaviors related to rearing (lifting up), grooming (self-cleaning) and number of crossings. In other words, it did not change the behavioral reactions, since there were no significant differences between the control, estragole and Es/β-CD groups at the dose of 60 mg/kg.

### 2.3. Evaluation of Peripheral and Central Antinociceptive Action of Es and Es/β-CD

#### 2.3.1. Abdominal Writhing Induced by 0.6% Acetic Acid

**Figure 2:**
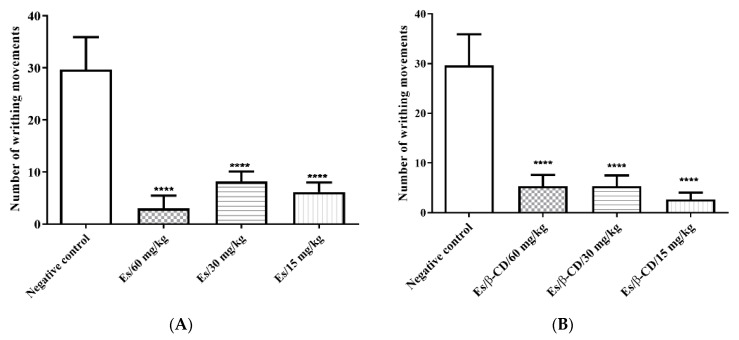
(**A**) Antinociceptive effect of estragole (Es) (60, 30 and 15 mg/kg) in the abdominal writhing test induced by acetic acid. (**B**) Antinociceptive effect of complex of estragole in β-CD (60, 30 and 15 mg/kg) in the abdominal writhing test induced by acetic acid, for (n = 6/group). The values show the arithmetic mean ± S.E.M (Standard Error of the Mean). One-way ANOVA followed by Tukey’s test (**** *p* < 0.0001, when compared to the negative control group).

In the abdominal writhing test, the groups treated with Es and Es/β-CD at doses of 60, 30 and 15 mg/kg showed a reduction of 89.89; 72.48 and 79.22%; and 82.03; 82.03 and 91.02% respectively, when compared to the negative control (Figure 2A,B).

#### 2.3.2. 2.5%. Formalin Test

**Figure 3:**
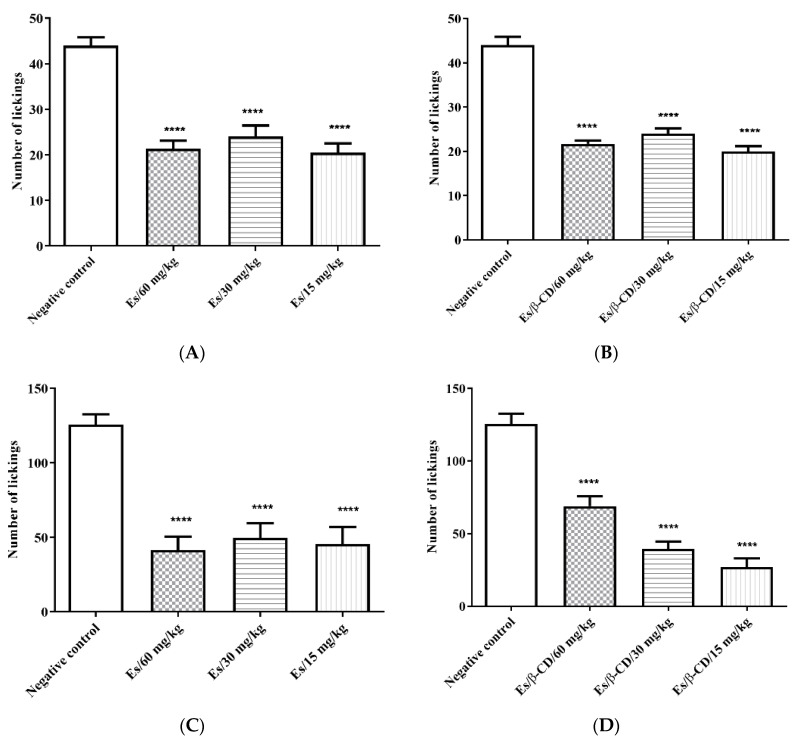
Evaluation of antinociceptive effect of estragole and Es/β-CD (60, 30 and 15 mg/kg) in the Formalin test. Effect in the neurogenic phase of Es (**A**) and Es/β-CD (**B**) and inflammatory phase to of Es (**C**) and Es/β-CD (**D**). Values show the mean ± S.E.M (Standard Error of the Mean) for (6 = n/groups). One-way ANOVA followed by Tukey’s test (**** *p* < 0.0001, when compared to the negative control group).

In the evaluation of the first phase of the Formalin assay, all the doses treated with the estragole and the Es/β-CD (60, 30 and 15 mg/kg) showed a significant reduction in paw licking time compared to the control group, at 51.53; 45.46; 53.41%, and 50.72; 45.46 and 54.56%, respectively (Figure 3A,B). In the evaluation of the second phase, all groups treated with Estragole and Es/β-CD (60, 30 and 15 mg/kg) showed a reduction in paw licking time of 66.94; 60.42 and 63.75%, and 45.16; 68.53 and 78.49%, respectively, compared to the negative control group (Figure 3C,D).

#### 2.3.3. Hot Plate Test

**Figure 4:**
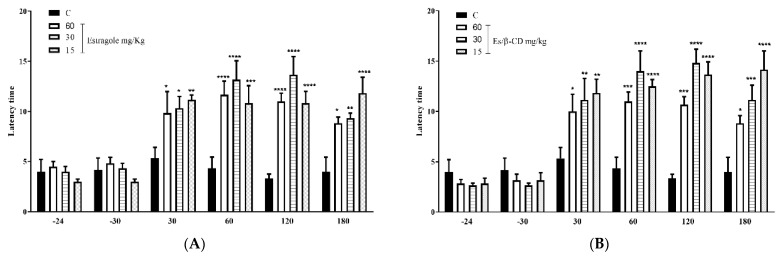
Antinociceptive effect of estragole (**A**) and Es/β-CD (**B**) (60, 30 and 15 mg/kg) in the hot plate test. Values show the mean ± S.E.M (Standard Error of the Mean) for (6 = n/groups). Two-way ANOVA followed by Tukey’s test (* *p* < 0.05; ** *p* < 0.01; *** *p* < 0.001; **** *p* < 0.0001, when compared to the negative control group).

In the hot plate test, the oral treatments (p.o.) with Es (60, 30 and 15 mg/kg. p.o.) significantly increased the animals’ permanence time on the plate by 99.8; 99.79; and 99.8% respectively (Figure 4A). Es/β-CD (60, 30 and 15 mg/kg) showed an increase of 99.82; 99.78; and 99.77% (Figure 4B), respectively, when compared to the control group in the time interval of 30 to 180 min.

#### 2.3.4. Tail Flick Test

**Figure 5:**
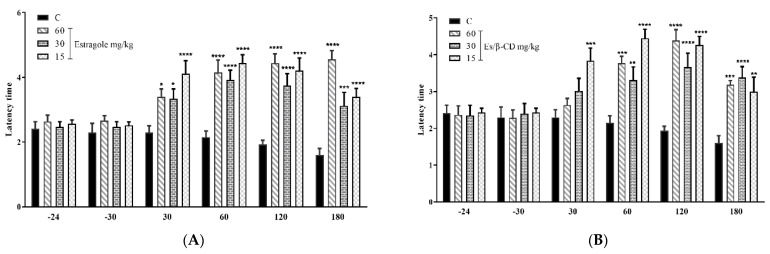
Antinociceptive effect of estragole (**A**) and Es/β-CD (**B**) (60, 30 and 15 mg/kg) in the tail flick test. Values show the mean ± S.E.M (Standard Error of the Mean) for (6 = n/groups). Two-way ANOVA followed by Tukey’s test. (* *p* < 0.05; ** *p* < 0.01; *** *p* < 0.001; **** *p* < 0.0001, when compared to the negative control group).

In the tail flick test, treatments with Es (60, 30 and 15 mg/kg. p.o.) significantly increased the tail flick time at 99.83; 99.85; and 99.83% (Figure 5A). The treatments with Es/β-CD (60, 30 and 15 mg/kg. p.o.), promoted an increase of 99.85; 99.86 and 99.84% (Figure 5B), respectively, when compared to the control group in the time interval of 30 to 180 min.

#### 2.3.5. Mechanical Hypernociception Pressure Test—Von Frey

**Figure 6:**
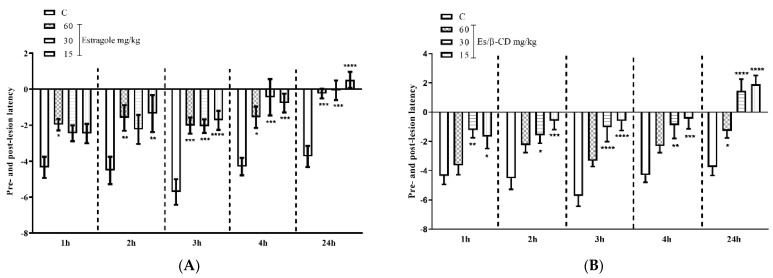
Antinociceptive effect of estragole (**A**) and Es/β-CD (**B**) (60, 30 and 15 mg/kg)in the von Frey test,. Values show the mean ± S.E.M (Standard Error of the Mean) for (6 = n/groups). Two-way ANOVA followed by Tukey’s test (* *p* < 0.05; ** *p* < 0.01; *** *p* < 0.001; **** *p* < 0.0001, when compared to the negative control group).

In the von Frey assay, treatments with Es (60, 30 and 15 mg/kg), by oral route, significantly reduced the pain threshold at 67.30; 67.92 and 74.29% and with Es/β-CD (60, 30 and 15 mg/kg), at 43.47; 85.68 and 93.81%, respectively. Thus, demonstrating the antinociceptive activity of estragole and Es/β-CD when compared to the control group in the interval and 1, 2, 3, 4 and 24 h after the formalin injection (Figure 6A,B).

### 2.4. Evaluation of Pain Signaling Pathways and their Interactions in the Antinociceptive Response of Estragole and Es/β-CD, (Opioid, Cholinergic, Nitric Oxide, A-Adrenergic, Dopaminergic, Adenosinergic, Glutamatergic, Cyclic Guanosine Monophosphate (cGMP), and Vanilloid Pathways)

#### 2.4.1. Action on the Opioid System

**Figure 7:**
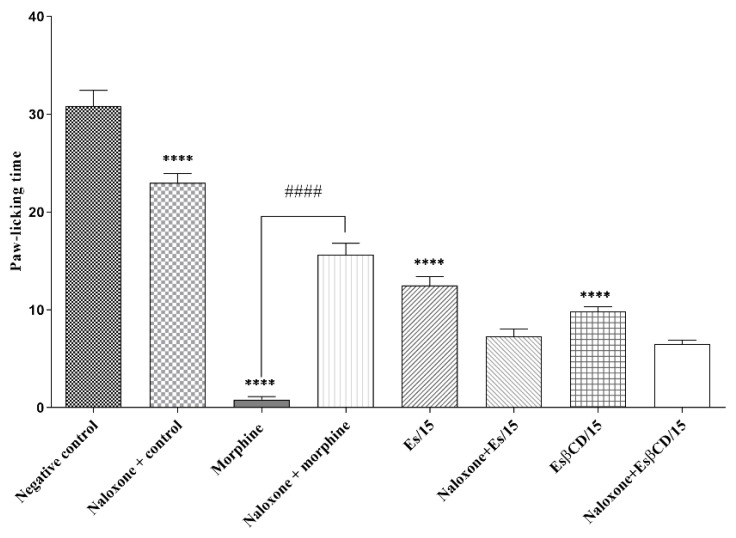
Antinociceptive response of estragole and Es/β-CD (15 mg/kg) in the opioid signaling pathway. Values show the mean ± S.E.M (Standard Error of the Mean) for (6 = n/group). One-way (ANOVA) followed by Tukey’s test (**** *p* < 0.0001, when compared to the negative control group). (#### *p* < 0.0001, when comparing the antagonist + agonist vs. agonist.

Treatments with Es and Es/β-CD, (p.o.) and morphine (pathway-specific agonist), (s.c.), significantly reduced the paw-licking time of animals after intraplantar formalin injection (0–5 min) at 59.45; 68.10 and 97.30%, respectively, when compared to the negative control group. There was no statistically significant difference when the animals in the Es and Es/β-CD groups received pre-treatment with naloxone (a specific inhibitor of the opioid pathway), thus suggesting that both Es and Es/β-CD do not act on the opioid signaling pathway. (Figure 7)

#### 2.4.2. Action on the Cholinergic System

Treatments with Es and Es/β-CD (p.o.) and acetylcholine (pathway-specific agonist) (s.c.) significantly reduced the paw-licking time of animals after intraplantar formalin injection (0–5 min) at 38.52; 52.39 and 66.68%, respectively, when compared to the negative control group.

**Figure 8:**
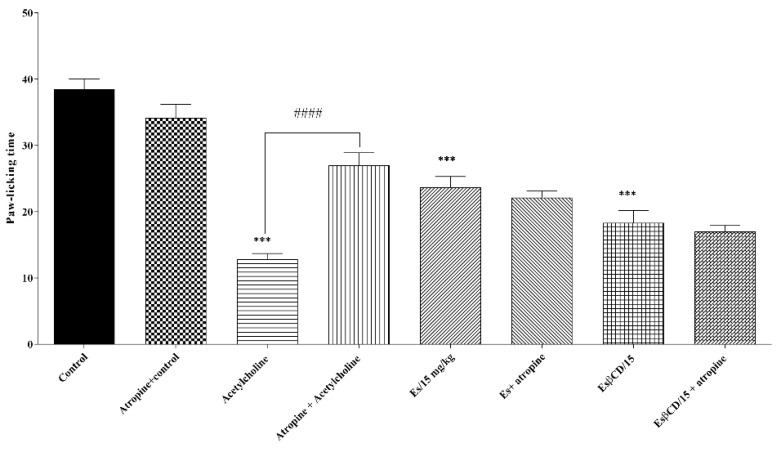
Antinociceptive response of estragole and Es/β-CD (15 mg/kg) in the cholinergic system signaling pathway. Values show the mean ± S.E.M (Standard Error of the Mean) for (6 = n/group). One-way (ANOVA) followed by Tukey’s test (significance of *p* < 0.0001—*** when compared to the negative control group). ####*p* < 0.0001, when comparing the antagonist + agonist vs. agonist).

However, there was no statistical difference when the animals in the estragole and Es/β-CD groups received pre-treatment with atropine (specific inhibitor of the cholinergic pathway), thus suggesting that both estragole and Es/β-CD are not acting on the cholinergic system signaling pathway. (Figure 8)

#### 2.4.3. Action on the Nitric Oxide Pathway

The treatments with Es and Es/β-CD (15 mg/kg), by oral route and L-NOARG (pathway agonist), intraperitoneally, significantly reduced the paw-licking time of the animals, after the intraplantar injection of formalin (0–5 min) at 64.97; 67.29 and 63.59%, respectively, when compared to the negative control group.

**Figure 9:**
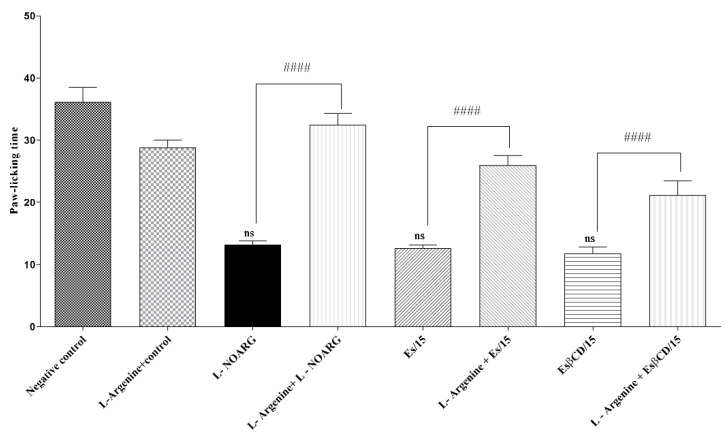
Antinociceptive response of estragole and Es/β-CD (15 mg/kg) in the nitric oxide signaling pathway. Values show the mean ± S.E.M (Standard Error of the Mean) for (6 = n/group). One-way (ANOVA) followed by Tukey’s test, when compared to the negative control group). (#### *p* < 0.0001, when comparing antagonist + agonist vs. agonist; estragole alone vs. atropine + estragole; Es/β-CD alone vs. atropine + Es/β-CD; ns – no significant.

However, when evaluating the animals of the Es and Es/β-CD groups, which received the pre-treatment with L-Arginine (pathway inhibitor), a process of reversal of the antinociceptive effect was observed, suggesting that both Es and Es/β-CD act on the nitric oxide signaling pathway (Figure 9)

#### 2.4.4. α-2 Adrenergic Receptor Activity

Treatments with oral Es and Es/β-CD and intraperitoneal clonidine (pathway-specific agonist) significantly reduced the animals’ paw-licking time after the intraplantar formalin injection (0–5 min) at 55.10; 51.68 and 90.69%, respectively, when compared to the negative control group.

**Figure 10:**
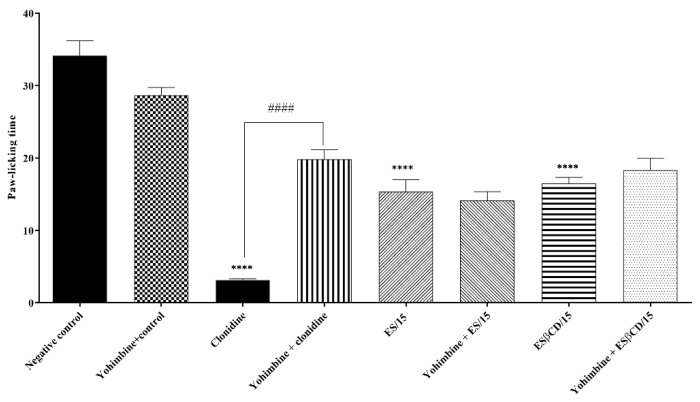
Antinociceptive response of estragole and Es/β-CD (15 mg/kg) in the α-2 adrenergic system signaling pathway. Values show the mean ± S.E.M (Standard Error of the Mean) for (6 = n/group). One-way (ANOVA) followed by Tukey’s test (**** *p* < 0.0001, when compared to the negative control group). (#### *p* < 0.0001, when comparing antagonist + agonist vs. agonist).

However, when evaluated, there was no statistical difference in the animals from the Es and Es/β-CD groups, which received pre-treatment with yohimbine (a specific inhibitor of the opioid pathway). This result suggests that both Es and Es/β-CD are not acting on the α-2 adrenergic system signaling pathway (Figure 10).

#### 2.4.5. Action on the Dopaminergic System

The treatments with Es and Es/β-CD (15 mg/kg), through the oral route, significantly reduced the paw-licking time of the animals, after the intraplantar injection of formalin (0–5 min) at 66.34 and 48.08%, respectively, when compared to the negative control group.

**Figure 11:**
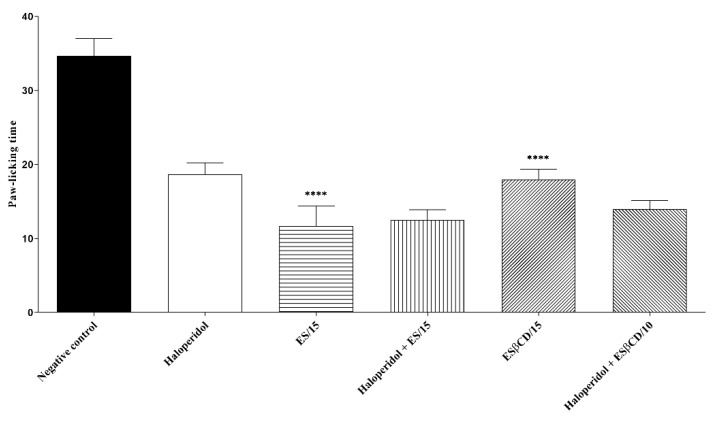
Antinociceptive response of estragole and Es/β-CD (15 mg/kg) in the dopaminergic signaling pathway. Values show the mean ± S.E.M (Standard Error of the Mean) for (6 = n/group). One-way (ANOVA) followed by Tukey’s test (**** *p* < 0.0001, when compared to the negative control group).

However, there was no statistical difference when the animals in the Es and Es/β-CD groups received pre-treatment with haloperidol (non-specific inhibitor of the pathway), thus suggesting that both estragole and Es/β-CD do not have any activity in the dopaminergic signaling pathway (Figure 11).

#### 2.4.6. Action on the Adenosinergic System

Treatments with Es and Es/β-CD (15 mg/kg/p.o.) significantly reduced the paw-licking time of animals after the intraplantar formalin injection (0–5 min) at 63.03 and 49.47%, respectively, when compared to the negative control group.

**Figure 12:**
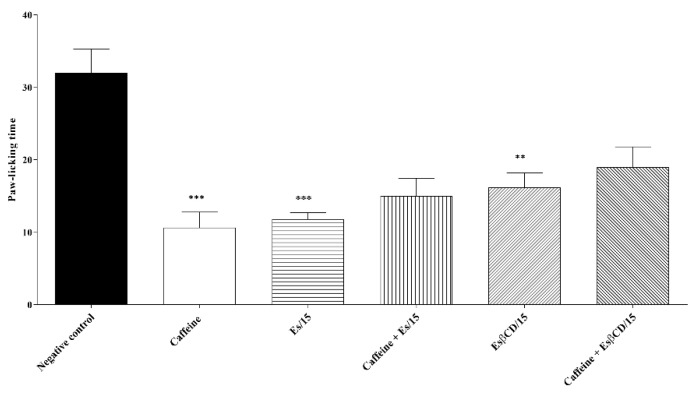
Antinociceptive response of estragole and Es/β-CD (15 mg/kg) in the adenosinergic signaling pathway. Values show the mean ± S.E.M (Standard Error of the Mean) for (6 = n/group). One-way (ANOVA) followed by Tukey’s test (*** *p* < 0.001; ** *p* < 0.01, when compared to the negative control group).

There was no statistical difference when the animals in the Es and Es/β-CD groups received pretreatment with caffeine (non-specific inhibitor of the pathway), thus suggesting that both Es and Es/β-CD do not have any activity on the adenosinergic signaling pathway (Figure 12).

#### 2.4.7. Action on the Glutamatergic Signaling Pathway

**Figure 13:**
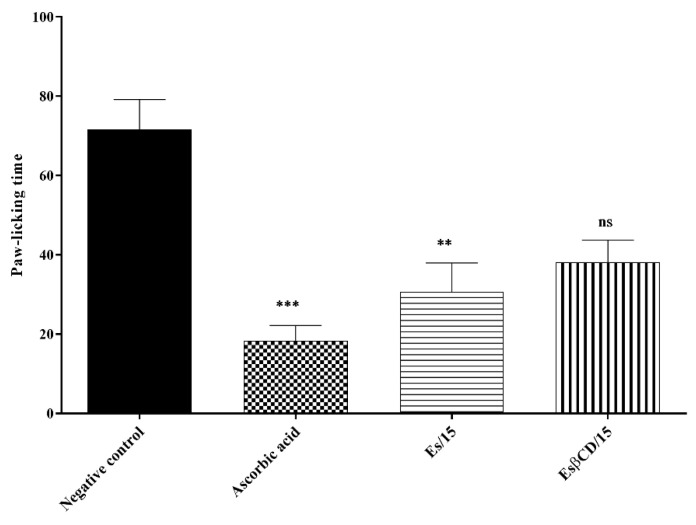
Antinociceptive response of estragole and Es/β-CD (15 mg/kg) in the glutamatergic signaling pathway. Values show as the mean ± S.E.M (Standard Error of the Mean) for (6 = n/group). One-way (ANOVA) followed by Tukey’s test (ns – no significant; ** *p* < 0.01; *** *p* < 0.001, when compared to the negative control group).

Treatments with Es and Es/β-CD (15 mg/kg), through the oral route, significantly reduced the paw-licking time of the animals, after the intraplantar injection of glutamate (an inflammatory agent specific to the glutamatergic pathway), at 57.21 and 46.74%, respectively, when compared to the negative control group. These results demonstrate the significant antinociceptive effect of the compounds on the glutamatergic signaling pathway (Figure 13).

#### 2.4.8. Action in the Cyclic Guanosine Monophosphate (cGMP) Signaling Pathway

The treatments with Es and Es/β-CD (15 mg/kg), through the oral route, and methylene blue, (i.p.), significantly reduced the paw-licking time of the animals, after the intraplantar injection of glutamate (specific inflammatory agent of the glutamatergic pathway), (0–15 min) at 24.70 and 97.32%, respectively, when compared to the negative control group.

**Figure 14:**
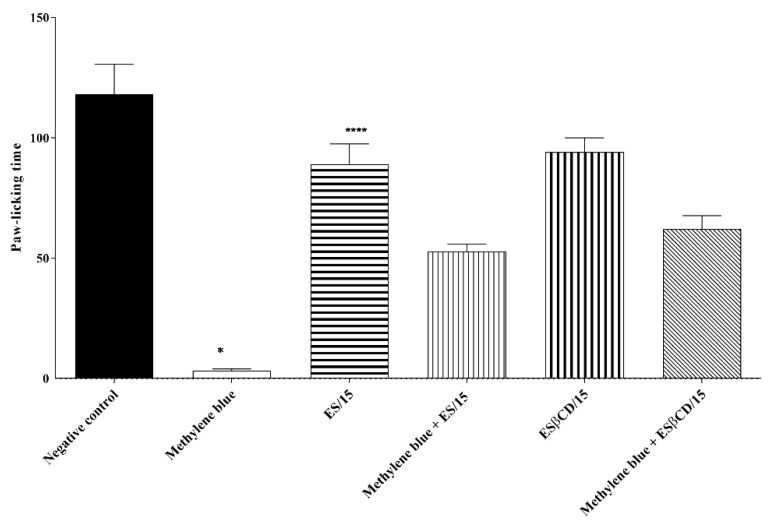
Antinociceptive response of estragole and Es/β-CD (15 mg/kg) in the cyclic guanosine monophosphate (cGMP) signaling pathway. Values show the mean ± S.E.M (Standard Error of the Mean) for (6 = n/group). One-way (ANOVA) followed by Tukey’s test (* *p* < 0.05; **** *p* < 0.0001, when compared to the negative control group).

Based on the results, it can be observed that there was no statistical difference in relation to the animals in the Es and Es/β-CD groups that received the pre-treatment with methylene blue (pathway-specific inhibitor), thus suggesting that estragole has an effect on the cyclic guanosine monophosphate signaling pathway (Figure 14).

#### 2.4.9. Action on the Vanilloid System

**Figure 15:**
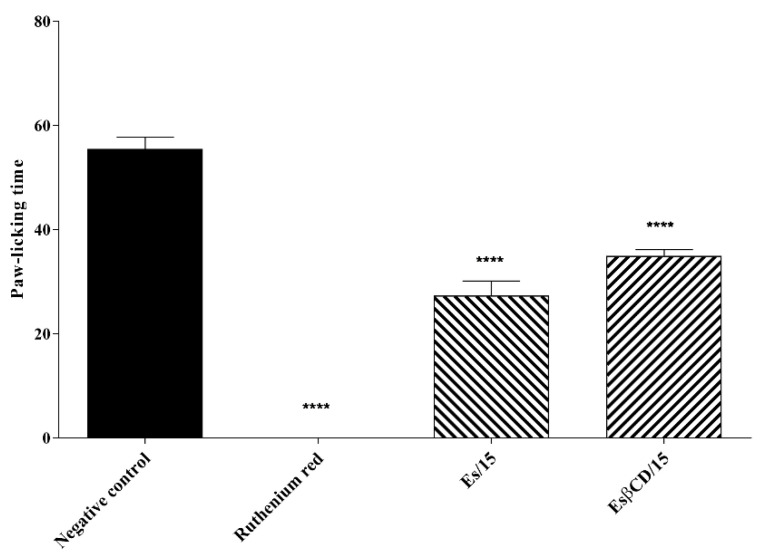
Antinociceptive response of estragole and Es/β-CD (15 mg/kg) in the vanilloid signaling pathway. Values show the mean ± S.E.M (Standard Error of the Mean) for (6 = n/group). One-way (ANOVA) followed by Tukey’s test (**** *p* < 0.0001; when compared to the negative control group).

Treatments with Es and Es/β-CD (15 mg/kg), through the oral route and ruthenium red (non-specific TRP antagonist) intraperitoneally, significantly reduced the paw-licking time of the animals after intraplantar capsaicin injection (specific inflammatory agent of the vanilloid pathway), (0–5 min) at 50.75; 36.94 and 100%, respectively, when compared to the negative control group. These results demonstrate the significant antinociceptive effect of the tested substances and prove their action on the vanilloid pathway (Figure 15).

## 3. Discussion

The present study, for the first time, provides a detailed analysis of the antinociceptive effect of Es and Es/β-CD, as well as the pain signaling pathways involved in pain response in animal models. The physicochemical analysis of Es/β-CD using the FT-IR technique proved to be effective, allowing the confirmation of the Es/β-CD complexation through the displacement of the bands, which showed common and characteristic peaks of Es, as well as β-CD, with a strong similarity (Figure 1A–C).

In a study carried out by Fonseca et al. [20], it was also possible to confirm the evidence of the inclusion complex formation between Es and β-CD through the ATR-FTIR method, clearly demonstrating the encapsulation of Es with β-CD, suggesting that complexation with these excipients is an effective method to improve the stability of substances.

In the models used to evaluate the effect of Es and Es/β-CD on the central nervous system, the treatments did not promote any changes in the evaluated parameters. Thus, the data presented herein suggest that the nociceptive actions of Es and Es/β-CD do not seem to be associated with unspecific central actions, as they did not significantly influence behaviors related to motor coordination (rota-rod), the number of crossings, self-cleaning (grooming) and vertical exploration of the mice (open field test). This places Es among the compounds that are favorable to a possible therapeutic application for the treatment of pain.

Literature data show that estragole exhibited a dose-dependent cytotoxic activity and did not show cytotoxicity to RAW 264.7 cells at doses of 674 µM [21]. The cytotoxicity assay for the estragole showed cell growth inhibition (IC50) values were 2280, 2684, and 267 µg /mL, respectively. The cell lines selected for in vitro analysis were human hepatoma cells (HepG2, SigmaAldrich, St. Louis, MI, USA), human cervical carcinoma cells (HeLa, Sigma-Aldrich), and peritoneal murine macrophages obtained from mice (Mus musculus) [22]. Other studies also evidenced that estragole exhibited low acute toxicity in vivo at single doses of up to 5.0 g/kg. We have published before that non-clinical acute toxicity tests with estragole, the animals treated with the dose of 625 mg/kg/v.o. showed no clinical signs of toxicity [23], and the LD_50_ value was determined with 1867.25 ± 310 mg/kg (v.o.) [24]. Based on acute toxicity tests, we have concluded that estragole is safe for animal use in assayed doses.

Regarding the antinociceptive effect, treatments with Es and Es/β-CD significantly reduced the number of abdominal writhing movements at all tested doses. However, Es/β-CD showed a more significant action than Es alone in this model. Based on the data presented for Es/β-CD, it was possible to observe that the lowest doses of the complex showed the best results, which confirms the more robust antinociceptive effect of the complex inclusion when related to isolated monoterpenes. The inclusion of substances in cyclodextrins has been studied, as they act to increase the improve stability, reduce the volatility, toxic effect, solubility, permeability and chemical stability of several volatile compounds and products [25].

The formalin test is a classic model for investigating the potential of analgesic substances, being characterized in two phases. The first phase (neurogenic pain) is associated with stimulation of type C and Aδ afferent fibers and promotes the release of excitatory amino acids, substance P, nitric oxide, and others. The second phase (inflammatory pain) involves the release of several pro-inflammatory chemical mediators, which include histamine, serotonin, bradykinin and prostaglandins [26].

Pre-treatment with Es and Es/β-CD at all doses in the formalin test showed an antinociceptive effect in both phases. In the study by Rodrigues et al., (2016) pre-treatment with estragole significantly reduced the acute and chronic inflammatory process, with this action being attributed to the inhibition of chemical mediators, vascular permeability and leukocyte migration. It is worth noting that the antinociceptive effect of Es and Es/β-CD in the formalin test corroborates the abdominal writhing test in this study, where a more significant effect was observed with Es/β-CD at its lowest dose when compared to Es alone.

The types of nociceptive stimuli (electrical, thermal, mechanical, or chemical) that have been used in different pain models are likely to more closely mimic acute clinical pain that affect supraspinal and spinal components [27]. In the tail flick, hot plate, and von Frey tests that act at the level of central receptors, it was possible to observe the action of Es and Es/β-CD in the reduction of pain perception caused by the stimuli generated in each assay. These results corroborate the effect observed in the writhing and formalin tests, suggesting that Es and Es/β-CD have centrally-acting antinociceptive activity. Recent previous studies have demonstrated the antinociceptive activity of monoterpenes, including 1,8-cineole [28] and geraniol [29].

From the confirmation of the antinociceptive action of Es and Es/β-CD, this study focused on investigating the involved signaling pathways using the lowest effective dose of the previous assays: 15 mg/kg of Es and Es/β-CD. The analyzed pathways were: opioid, cholinergic, nitric oxide, α2 adrenergic, dopaminergic, adenosinergic, glutamatergic, cyclic guanosine monophosphate and vanilloid pathways. Based on the tests, both Es and Es/β-CD demonstrated activity in the systems: nitric oxide, glutamatergic, guanosine monophosphate and vanilloid pathways.

As for the L-arginine/nitric oxide/cGMP pathway, Es and Es/β-CD had their antinociceptive effects reversed when associated with the pathway antagonist (L-arginine), demonstrating a possible participation in the antinociceptive effect of both substances. In inflammatory pain, NO is derived from migrating cells, such as neutrophils [30]. In the present study, Es and Es/β-CD may possibly be acting on the recruitment of neutrophils, and their antinociceptive action can be explained by the modulation of the migration of these cells, causing a reduction in tissue damage and, consequently, in nociception.

Regarding the glutamatergic system, Es and Es/β-CD showed a good response in reducing paw-licking time when compared to the control and ascorbic acid, suggesting a possible participation of Es and Es/β-CD compounds in the tested pathway. Glutamate receptors are located in the central and peripheral nervous system, being involved in the sensation and transmission of pain [31]. Ascorbic acid promotes extracellular glutamate accumulation, involving glutamate uptake inhibition; it also increases the activity of NMDA receptors, promoting a decrease in glutamate-stimulated levels [32].

Transient receptor potential (TRP) channels constitute a large family of ion channels capable of being activated in different ways; the TRPV subfamily has six members that can be broadly divided into low selectivity cation channels and channels that show high selectivity to Ca2+ [33,34]. Ruthenium red is a non-competitive antagonist with the capacity to block transmembrane and mitochondrial Ca2+ sequestration and inhibit capsaicin-mediated excitatory effects on sensory neurons and peripheral nociceptors; this fact justifies its antagonistic effect on capsaicin-induced nociceptive response [35].

As for the vanilloid system, treatments with Es and Es/β-CD significantly reduced the paw-licking time of the animals after the intraplantar capsaicin injection, demonstrating the significant antinociceptive effect of the tested substances and proving their possible participation in the vanilloid pathway.

## 4. Materials and Methods

### 4.1. Assessed Substances and Drugs

The substances were obtained from Sigma-Aldrich (St. Louis, MO, USA). All substances were prepared immediately before oral, intraperitoneal, intraplantar and subcutaneous administration, according to the animal weight (0.1 mL/10 g of body mass) and specific protocols.

### 4.2. Preparation of Inclusion Complexes in β-cyclodextrins (β-CD)

The β-CD inclusion complex was prepared by the co-evaporation and precipitation method, according to the procedures described by [36], with modifications. Approximately 7 g of β-CD were dissolved in 20 mL of distilled water and acetone (3:1 v/v) at 40 °C for 30 min. Then, the estragole in hydro-acetone solutions of β-CD was added, with continuous stirring for 1 h at 300 rpm at 37 °C. After that, the mixture was sonicated for 10 min at 4 °C to decrease particle size and incubated for 12 h at 4 °C. Afterwards, the mixture was left to stir on the magnetic stirrer for 36 h at 35 °C at 300 rpm, and after the stirring time, the mixture was left for 24 h in the freezer. The samples were lyophilized under vacuum at 40 °C for 36 h.

### 4.3. Characterization of the Inclusion Complexes Es/β-CD by Infrared Spectroscopy Measurement

The chemical characterization of the estragole inclusion complex in β-cyclodextrin (Es/β-CD), as well as of the β-cyclodextrin (β-CD) and estragole alone was carried out using the infrared spectroscopy technique. The attenuated total reflection Fourier-transform infrared (ATR-FTIR) absorbance spectra were obtained using an Agilent spectrometer, model CARY 660 FT-IR, with the results being processed using the software for infrared spectroscopy (OriginPro v.8.5). The ATR-FTIR spectrum was recorded at room temperature, with a spectral resolution of 4 cm^−1^ which performed 32 scans in the wave number region from 4000 cm^−1^ to 600 cm^−1^.

### 4.4. Animals

Male and female Swiss mice (*Mus musculus*) weighing between 20 and 30 g, obtained from the Animal Containment Unit of Universidade Regional do Cariri, were used in the study. The animals were housed with food and water *ad libitum* (Labina, Purina, Brazil) in a room with a controlled temperature of 24 ± 2 °C, with a 12 h light/dark cycle. Prior to the experiments, the animals were kept in the Laboratory of Pharmacology and Molecular Chemistry (LFQM) of Universidade Regional do Cariri-URCA—for a period of 24 h for acclimatization. The study was carried out in accordance with the recommendations of the National Council for the Control of Animal Experimentation (CONCEA) and the protocols were approved by the Ethics Committee on Animal Use of the Universidade Regional do Cariri (CEUA N. 358/2019-2).

### 4.5. In Vivo Assay

To evaluate the antinociceptive effect, the following protocols were performed: abdominal writhing test induced by 0.6% acetic acid, 2.5% formalin test, hot plate test, tail flick test, plantar mechanical hyperalgesia test and von Frey test. The animals were divided into groups (n = 6) and treated with H_2_O (0.1 mL/10 g/p.o.), Es and Es/β-CD (60, 30 and 15 mg/kg/p.o.) in the formalin screening tests and abdominal writhing, hot plate, tail flick and von Frey; for the other tests, to evaluate the pain signaling pathways, the lowest effective dose of Es and Es/β-CD was used, being 15 mg/kg/p.o., as defined from previous screening protocols. To evaluate the pain signaling involved in the antinociceptive response, the opioid, cholinergic, nitric oxide, α–adrenergic 2, dopaminergic, adenosinergic, glutamatergic, cyclic guanosine monophosphate and vanilloid pathways were investigated.

### 4.6. Assessment of Estragole and Es/β-CD on the CNS

The evaluation of the influence of Es and Es/β-CD on the central nervous system was performed only with the highest effective dose of 60 mg/kg using the open field and rota-rod assays.

#### 4.6.1. Open Field

Swiss mice (n = 6) were treated with H_2_O (0.1 mL/10 g/p.o.), Es and Es/β-CD (60 mg/kg/p.o.). After 1 h (p.o.) the animals were individually placed in the open field for a period of 5 min, where their horizontal exploration behaviors (number of crossings), self-cleaning behavior (grooming) and vertical exploration (rearing) were recorded [37].

#### 4.6.2. Rota-rod

Swiss mice (n = 6) were selected and pre-trained with up to 3 sessions (1 min) 24 h before the treatment. The selected animals were divided into groups and treated respectively with H_2_O (0.1 mL/10 g/p.o.), Es and Es/β-CD (60 mg/kg/p.o.), and after 1 h (p.o.) the animals were placed on the rota-rod device for 1 min (16 rpm) and the number of falls was recorded [38].

### 4.7. Evaluation of Peripheral and Central Antinociceptive Action of Es and Es/β-CD

#### 4.7.1. Abdominal Writhing Induced by Acetic Acid (0.6%)

Swiss mice (n = 6) were treated with H_2_O (0.1 mL/10 g/p.o.), Es and Es/β-CD (60, 30 and 15 mg/kg). One hour (p.o.) after the treatments, the animals received glacial acetic acid PA (0.6%/0.1 mL/10 g/i.p.) diluted in water for injection. After the administration of acetic acid, the animals were placed under individual transparent glass funnels for 30 min and the number of abdominal writhing movements was cumulatively quantified and characterized by the contraction and rotation of the abdomen, followed by the extension of one or both hind legs [39].

#### 4.7.2. 2.5% Formalin Test

Swiss mice (n = 6) were treated with H_2_O (0.1 mL/10 g/p.o.), Es and Es/β-CD (60, 30 and 15 mg/kg). After 1 h (p.o.), the animals were injected with 20 μL of formalin (2.5%) in the right hind paw (subplantar space) and placed individually immediately afterwards under an inverted glass funnel, next to a mirror to facilitate the observation. The time was recorded in seconds, in which the animal licked, continued licking or biting the injected paw (“licking time’) during the first phase, attributed to the neurogenic phase (0–5 min) and the second characterized as the inflammatory phase (15–30 min) [40].

#### 4.7.3. Hot Plate Test

The Swiss mice (n = 6) were individually placed on a hot plate (52–54 °C ± 0.5 °C) and after obtaining two baseline values at 24 h and 30 min before the test, the mice were treated (p.o.) according to the groups: H_2_O (0.1 mL/10 g/p.o.), Es and Es/β-CD (60, 30 and 15 mg/kg). Subsequently, the response was evaluated 30, 60, 120 and 180 min after treatment administration, with the maximum contact time of the animal with the hot plate being held at 15 s (baseline cutoff time) and 30 s (test cutoff time) to avoid paw injuries. The nociceptive response was characterized by shaking the hind paws, licking or lifting the paw, or jumping on the plate [41]. To compare the effects over time, the percentage of each group was calculated using the average effect observed at times 30 up to 180 min.

#### 4.7.4. Tail Flick Test

Swiss mice (n = 6) were divided into 4 groups and placed in an apparatus containing a thermal light source and a mouse tail holder. Soon after that, two baseline values were obtained, 24 h and 30 min before the test. Afterwards, the mice were treated (p.o.) according to the groups: H_2_O (0.1 mL/10 g/p.o.), Es and Es/β-CD (60, 30 and 15 mg/kg). At the moment when the tail was placed in the apparatus, the noxious heat source was activated and the timer started, and the time until tail withdrawal was evaluated at 30, 60, 120 and 180 min after the treatments. A maximum latency period of 15 s was established to avoid tissue damage [42]. The results were expressed as mean ± standard error of mean of the latency time.

#### 4.7.5. Plantar Mechanical Hyperalgesia Test—Von Frey

Swiss mice (n = 6) were divided into 4 groups treated with H_2_O (0.1 mL/10 g/p.o.), Es and Es/β-CD (60, 30 and 15 mg/kg). After 60 min (p.o.) of the treatments, all animals were injected with 20 μL of formalin (2.5%) in the right hind paw. The animals were placed individually in glass boxes on an elevated surface and covered with wire mesh. Stimuli were applied with the von Frey anesthesiometer filament to the injected plantar surface. The device records the sufficient force in grams to remove the paw in contact with the filament [43]. Positive responses were those in which the animal performed withdrawal movements, followed by shivering after the mechanical stimulation. All tests were performed after obtaining two baseline measurements (with an interval of 24 h) [44]. After the formalin injection, each animal was evaluated after 1, 2, 3, 4 and 24 h. The results were expressed as the mean of the difference between the baseline value and that after the formalin injection (Δ), in the aforementioned time periods [45,46].

### 4.8. Evaluation of Pain Signaling Pathways and Their Interactions in the Antinociceptive Response of Es and Es/β-CD (Opioid, Cholinergic, Nitric Oxide, A-Adrenergic, Dopaminergic, Adenosynergic, Glutamatergic, Cyclic Guanosine Monophosphate (Cgmp), and Vanilloid Pathways)

#### 4.8.1. Action on the Opioid System

Swiss mice (n = 6) were divided into 8 groups, so that the first 4 groups were treated with H_2_O (0.1 mL/10 g/p.o.), opioid agonist-morphine (5 mg/kg s.c.), Es and Es/β-CD (15 mg/kg/p.o.) respectively, whereas the remaining 4 groups received naloxone-opioid antagonist (4 mg/kg i.p.) 15 min before the treatment. After 1 h (p.o.) or 30 min (s.c.) of the treatments, the animals were evaluated through the 2.5% formalin-induced nociception test (0–5 min) [39].

#### 4.8.2. Action on the Cholinergic System

Swiss mice (n = 6) were divided into 8 groups, where the first 4 groups were treated with H_2_O (0.1 mL/10 g/p.o.), acetylcholine (cholinergic agonist—1 mg/kg i.p.), Es or Es/β-CD (15 mg/kg/p.o.), while the other 4 groups were pre-treated with atropine (non-selective cholinergic antagonist 1 mg/kg/i.p.) 15 min before treatment with Es or Es/β-CD. Subsequently, 1 h (p.o.) or 30 min (i.p.) after the treatment, the animals were evaluated through the 2.5% formalin test (0–5 min) [47].

#### 4.8.3. Action on the Nitric Oxide Pathway

Swiss mice (n = 6) were divided into 8 groups, where the first 4 groups were treated with H_2_O (0.1 mL/10 g/p.o.), L-NG-nitroarginine (L-NOARG); 75 mg/kg, i.p.), Es and Es/β-CD (15 mg/kg/p.o.), while the other 4 groups were pre-treated with L-Arginine (NOS substrate 600 mg/kg/i.p.), 15 min before treatment with Es or Un/CD. Then, 1 h (p.o.) or 30 min (i.p.) after treatment, the animals were evaluated through the 2.5% formalin-induced nociception test (0–5 min) [48].

#### 4.8.4. α-2. Adrenergic Receptor Activity

Swiss mice (n = 6) were divided into 8 groups, where the first 4 groups were treated with H_2_O (0.1 mL/10 g/p.o.), clonidine (α-2 agonist 0.1 mg/kg/ i.p.), Es and Es/β-CD (15 mg/kg/p.o.), while the other 4 groups received yohimbine (α-2 antagonist—0.15 mg/kg i.p.) 15 min before treatment with Es or Es/β-CD. Then, 1 h (p.o.) or 30 min (i.p.) after treatment the animals were evaluated in relation to the 2.5% formalin test (0–5 min) [49].

#### 4.8.5. Action on the Dopaminergic System

Swiss mice (n = 6) were divided into 6 groups: the first 4 groups were treated with H_2_O (0.1 mL/10 g/p.o.), haloperidol (non-selective dopamine receptor antagonist 2 mg/kg i.p.), Es and Es/β-CD (15 mg/kg/p.o.), while the other 2 groups were pre-treated with haloperidol 15 min before treatment with Es or Es/β-CD. Then, 1 h (p.o.) or 30 min (i.p.) after treatment, the animals were evaluated in relation to the 2.5% formalin test (0–5 min) [50].

#### 4.8.6. Action on the Adenosinergic System

Swiss mice (n = 6) were divided into 6 groups: the first 4 groups were treated with H_2_O (0.1 mL/10 g/p.o.), caffeine (10 mg/kg i.p.), Es and Es/β-CD (15 mg /kg/p.o), while the other 2 groups were pre-treated with caffeine 15 min before treatment with Es or Es/β-CD. Then, 1 h (p.o.) or 30 min (i.p.) after treatment the animals were evaluated in relation to the 2.5% formalin test (0–5 min) [51].

#### 4.8.7. Action on the Glutamatergic Signaling Pathway

Swiss mice (n = 6) were divided into 4 groups treated with H_2_O (0.1 mL/10 g/p.o.), ascorbic acid (NMDA receptor antagonist 100 mg/kg i.p.), Es and ES/β-CD (15 mg/kg/p.o.). Then, 1 h (p.o.) or 30 min (i.p.) after treatment the animals were analyzed for 15 min in relation to the nociception induced by the intraplantar injection of 20 μL of buffered glutamate at 20 μmoL/paw, where the time the animal spent licking the paw was considered a parameter suggestive of pain [52].

#### 4.8.8. Action on the Cyclic Guanosine Monophosphate (cGMP) Signaling Pathway

To verify the involvement of cyclic guanosine monophosphate in the antinociception caused by the compounds under study, the animals were pre-treated with methylene blue (20 mg/kg/i.p.), a guanylate cyclase inhibitor, 15 min before the administration of Es and Es/β-CD (15 mg/kg/p.o.). In addition, other groups were treated with H_2_O alone (0.1 mL/10 g/p.o.), Es or Es/β-CD (15 mg/kg/p.o.) or methylene blue (20 mg/kg, i.p.). The nociceptive response was evaluated after 1 h (p.o.) or 15 min (i.p.) of the treatments, through the intraplantar injection of 20 μL of formalin solution and the paw licking time (0–5 min) was quantified [53].

#### 4.8.9. Action on the Vanilloid System

Swiss mice (n = 6) were divided into 4 groups, which were treated with H_2_O (0.1 mL/10 g/p.o.), ruthenium red (non-selective TRP antagonist 3 mg/kg i.p.) [54], Es and ES/β-CD (15 mg/kg/p.o.). Subsequently, 1 h (p.o.) or 30 min (i.p.) after treatment the animals were analyzed for 5 min in relation to the nociception induced by the intraplantar injection of 20 μL of capsaicin (TRPV1 receptor agonist) at 5.2 nmol/paw [55] where the time (seconds) during which the animal spent licking the paw was considered as behavior suggestive of pain.

### 4.9. Statistical Analysis

The results are presented as mean ± standard error of the mean (S.E.M), evaluated by one-way and two-way analysis of variance (ANOVA), using Tukey’s multiple comparison tests, and the calculations were performed using the statistical software GraphPad Prism (version 9.0), according to the values obtained in the tests. For all analyses, *p* < 0.05 was considered significant.

## 5. Conclusions

The present study showed that, regarding the activity on the central nervous system, estragole (Es) and estragole/β-cyclodextrin (Es/β-CD) did not alter exploratory or motor coordination activity, suggesting they do not have a depressant or excitatory action in the CNS. As for the antinociceptive effect, Es and Es/β-CD showed central and peripheral antinociceptive activity in the abdominal writhing model induced by acetic acid, formalin test, hot plate, tail flick and von Frey assay, with involvement of the nitric oxide, glutamatergic, guanosine monophosphate and vanilloid pathways. Importantly, Es/β-CD showed significant effects at lower doses than Es alone. The data of the present study suggest that Es and Es/β-CD may contribute to the formulation of new compounds for the treatment of pain. 

## Data Availability

The data presented in this study are available in the article.
